# V-shaped lymph node dissection in laparoscopic distal gastrectomy; new technique of intra-abdominal dissection and surgical outcomes

**DOI:** 10.1186/1477-7819-10-205

**Published:** 2012-09-29

**Authors:** Nobuhisa Matsuhashi, Narutoshi Nagao, Yoshinori Iwata, Sang-Woong Lee, Takaya Tokuhara, Chihiro Tanaka, Masahiko Kawai, Katsuyuki Kunieda, Kazuhiro Yoshida

**Affiliations:** 1 Department of Surgery, Gifu Prefectural General Medical Center, 1-1 Yanagido, Gifu City, Japan; 2 Department of General and Gastroenterological Surgery, Osaka Medical College, Osaka, Japan; 3 Department of Surgical Oncology, Gifu University School of Medicine, Gifu City, Japan

**Keywords:** Laparoscopic gastrectomy, Lymph node dissection

## Abstract

**Background:**

Recently, laparoscopic-assisted distal gastrectomy (LADG) has become popular for the treatment of early gastric cancer. Furthermore, the use of totally laparoscopic gastrectomy (TLG), a more difficult procedure than LADG, has been increasing in Japan. Laparoscopic-assisted distal gastrectomy is currently performed more frequently than laparoscopic distal gastrectomy (LDG) in hospitals in Japan.

**Method:**

Reconstruction after LDG is commonly performed extra-abdominally and lymph node dissection of the lesser curvature is performed at the same time. We have developed a new method of intra-abdominal lymph node dissection for the lesser curvature.

**Results:**

Our technique showed positive results, is easy to perform, and is reasonable in terms of general oncology theory.

**Conclusion:**

In oncological therapy, this technique could be a valuable surgical option for totally laparoscopic surgery.

## Background

We have developed a new method of intra-abdominal lymph node dissection for the lesser curvature, called V-shaped dissection. In this report, we present the outcome of our initial experience with this procedure.

### Patients

Fourteen patients with early gastric cancer patients who underwent totally laparoscopic gastrectomy (TLG) using our new method reported lesser curvature lymph node dissection from April 2011 to November 2011 at Gifu Prefectural General Medical Center, Japan. During the same period, 31 patients who underwent conventional distal gastrectomy (CDG) reported advanced gastric cancer combined with pathological lymph nodes in the lesser curvature.

## Method

At our Unit for Laparoscopic Gastrectomy, we are able to check for and confirm gastric cancer up to stages T1a(M), T1b(SM), N0, IA in preoperative diagnosis [1]. We are also able to check for distal gastrectomy indicated by distal and middle third gastric cancers in which tumor margins of at least 2 to 3 cm for early lesion can be taken.

However, proximal gastrectomy and total gastrectomy in total laparoscopic surgery are currently not included in our skill set at the unit.

**Figure 1:**
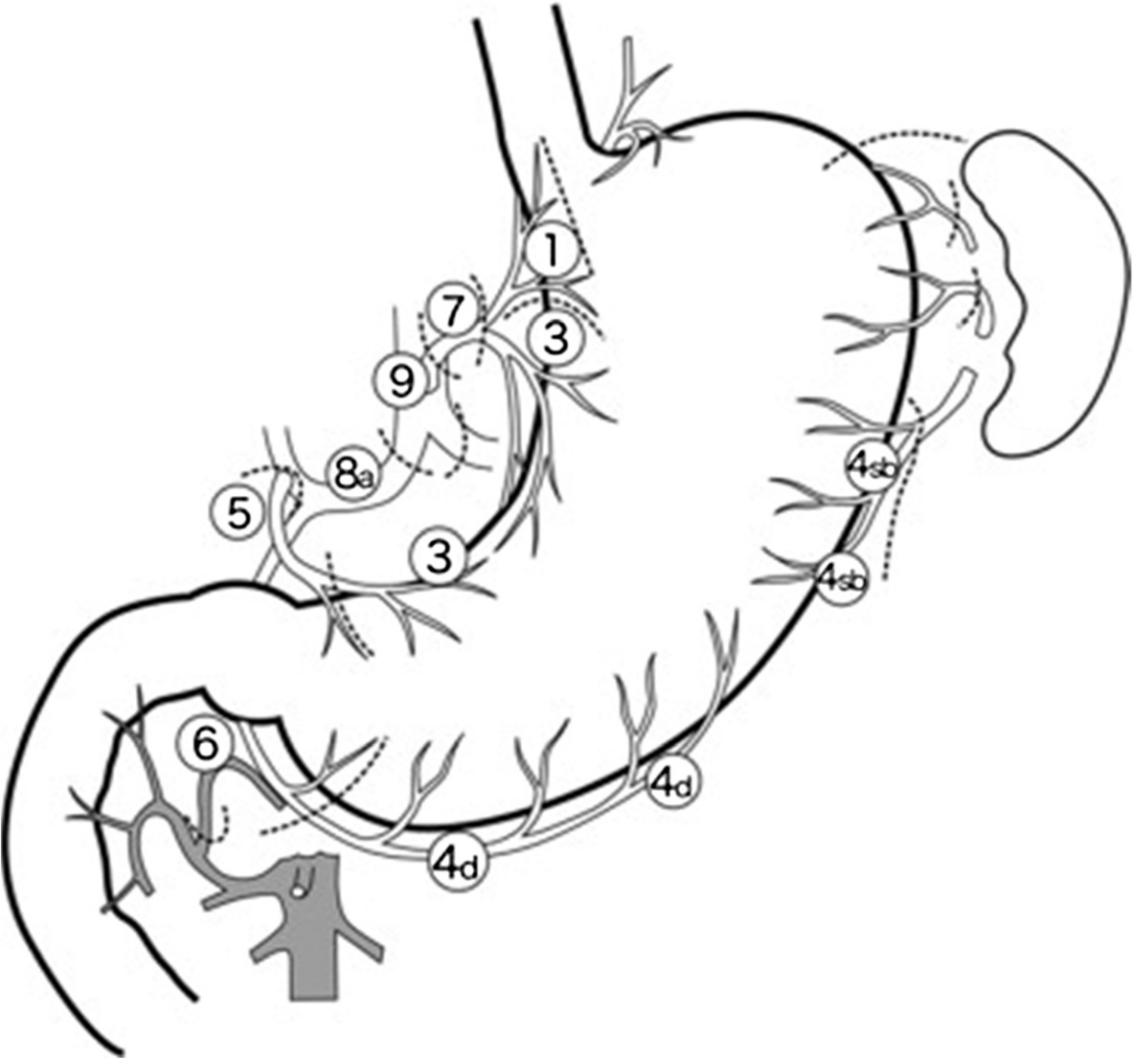
**Lymph node station**[1].

Lymph node dissection is performed depending on the endoscopic depth of invasion of the primary tumor and lymph node involvement with computed tomography. The preoperatively planned extent of lymphadenectomy was categorized as D1 (stages 1, 3, 4Sb, 4d, 5, 6, and 7), D1 + (D1 with stages 8a and 9), according to the latest Japanese treatment guidelines at our unit in November 2011 (Figure 1).

**Figure 2:**
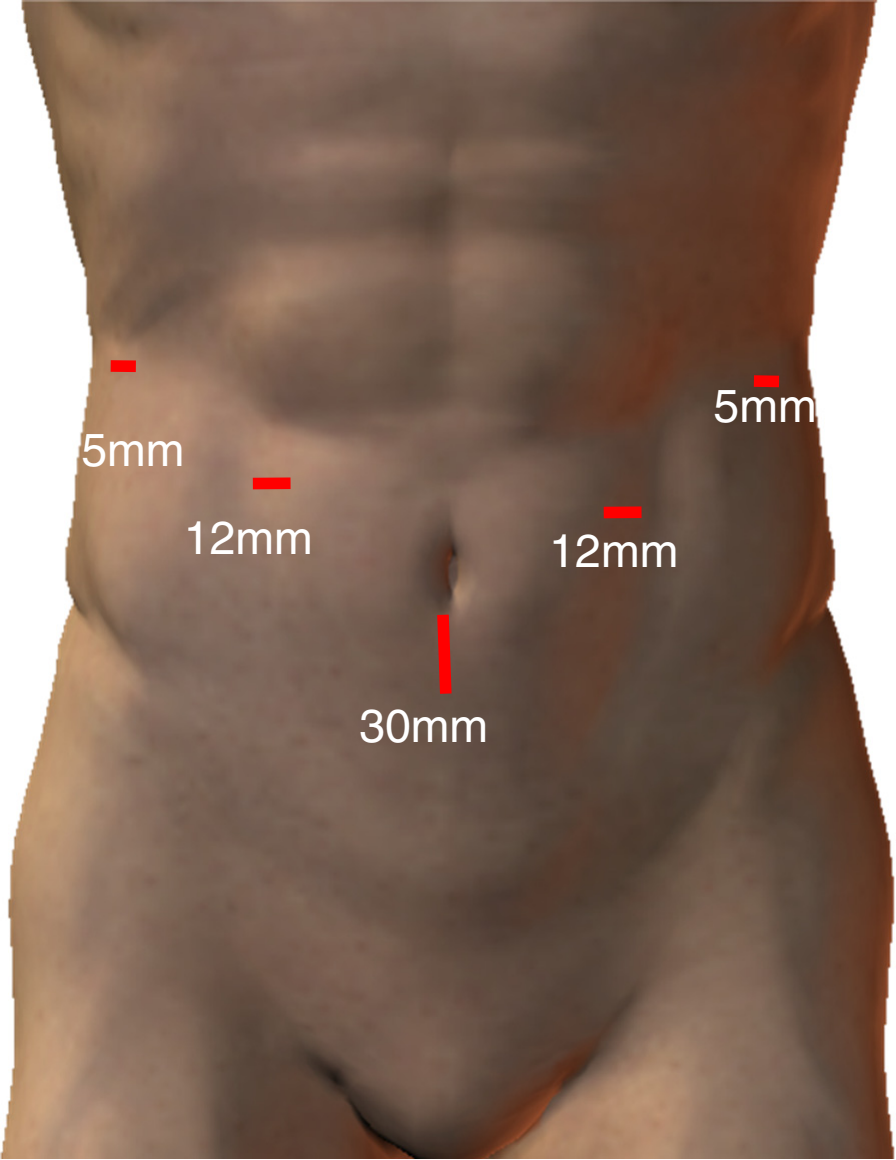
**Trocal placement.** Endoscopic liner stapler is inserted through the left lower port.

Under general anesthesia, the patient was placed in the reverse Trendelenburg’s position with the legs apart. Five trocars were placed, as shown in Figure 2. Laparoscopic distal gastrectomy (LDG) was performed with CO_2_ pneumoperitoneum.

Step 1: initially, we conducted a greater curvature procedure, identified, clipped and cut the left gastroepiploic artery and vein. This procedure was then performed on the right side.

Step 2: we moved to the patient’s left side and performed the greater omentectomy from the patient’s left side towards the patient’s right side, and identified the right gastroepiploic vein, which was clipped and cut. This procedure was then repeated for the right gastroepiploic artery on the patient’s left side.

Step 3: we performed a lesser curvature procedure, identified, clipped, and cut the right gastric artery and vein.

Step 4: the stomach was transected below the pylorus ring.

Step 5: we performed a procedure on the leg of the diaphragm, identified, clipped, and cut the left gastric vein and left gastric artery.

Sufficient lymphadenectomy was performed at each of the five steps.

**Figure 3:**
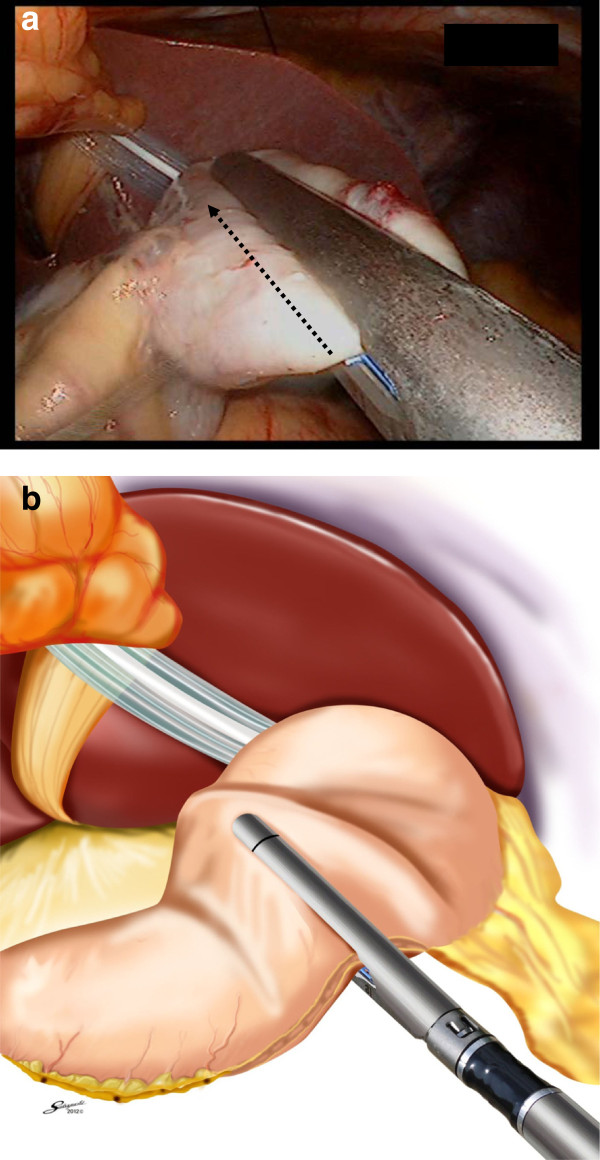
Insertion of a cartridge fork along the stomach from greater curvature.

Finally, we performed the new method of lymph node dissection in the intra-abdominal lesser curvature. We trimmed the greater curvature side. Using a schematic representation, we inserted the forks of the endoscopic linear stapler (Esheron Flex: ECR60B: Ethicon Endo-Surgery, Cincinnati) from the greater curvature to the lesser curvature (Figure 3).

**Figure 4:**
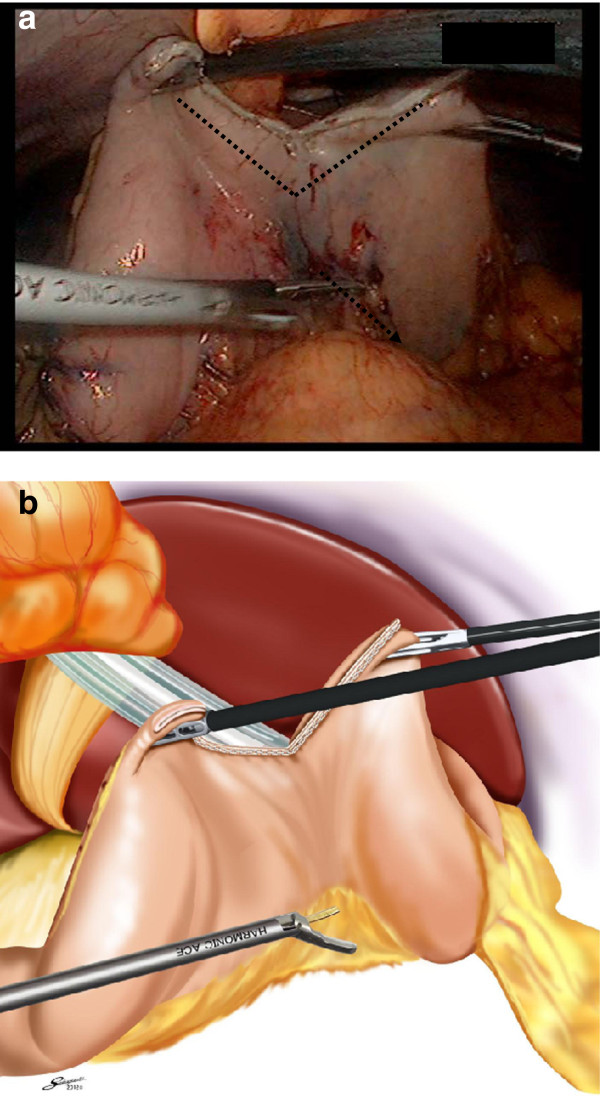
**At this point the separation of the half area is stopped.** The assistant operator lifts the stomach, to show the V shape.

**Figure 5:**
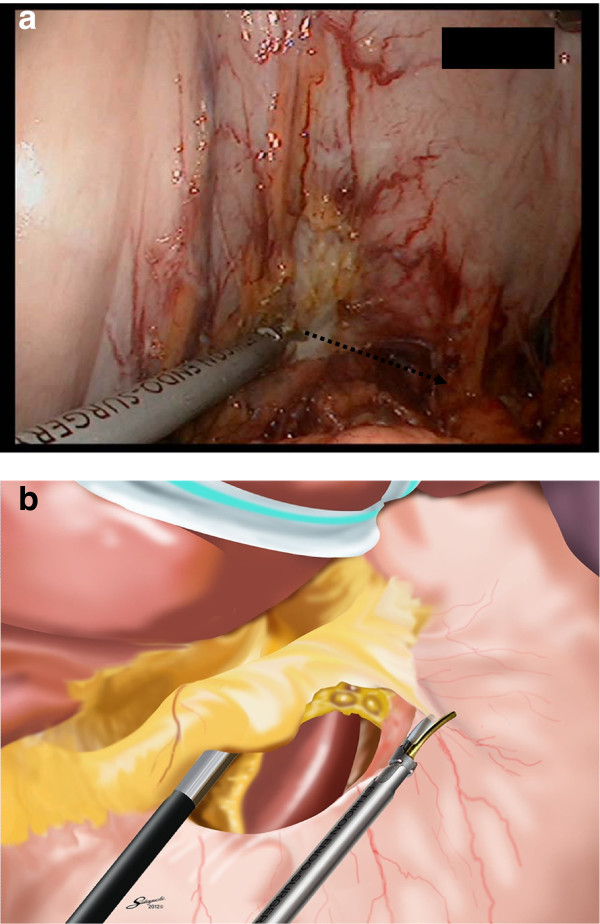
Operator’s forceps form a straight line on the axis against the ventral side of the lesser curvature.

**Figure 6:**
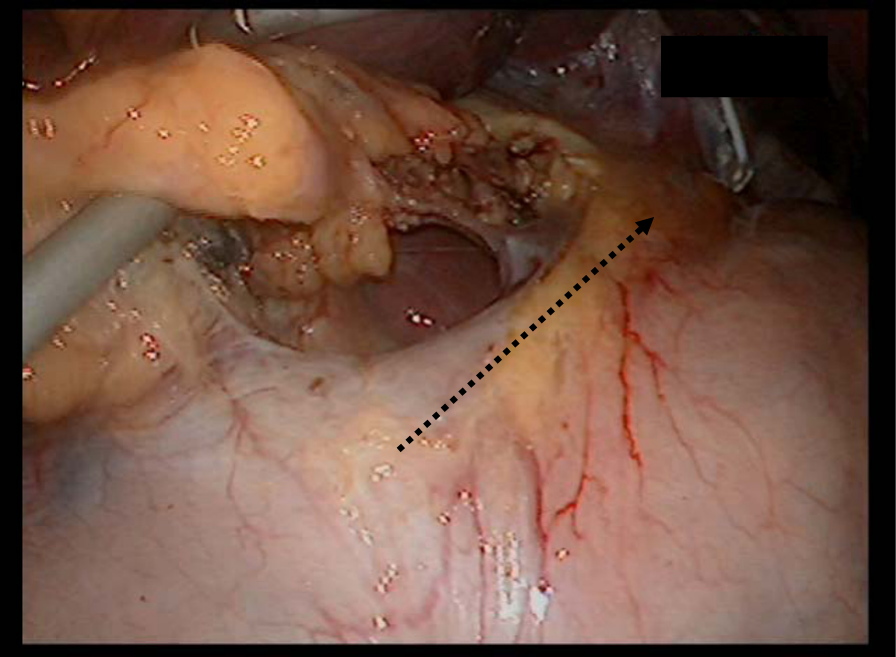
Operator’s forceps forma a straight line on the axis against the dorsal side of the lesser curvature.

At this point, separation of the halves is stopped. Additionally, the stomach begins to move towards the ventral side, catching each edge. The stomach now took on a separated V shape (Figure 4). The lymphadenectomy of the dorsal side ensued from the separation of the half area (Figure 5). In addition, the stomach returned to its normal position (Figure 6).

**Figure 7:**
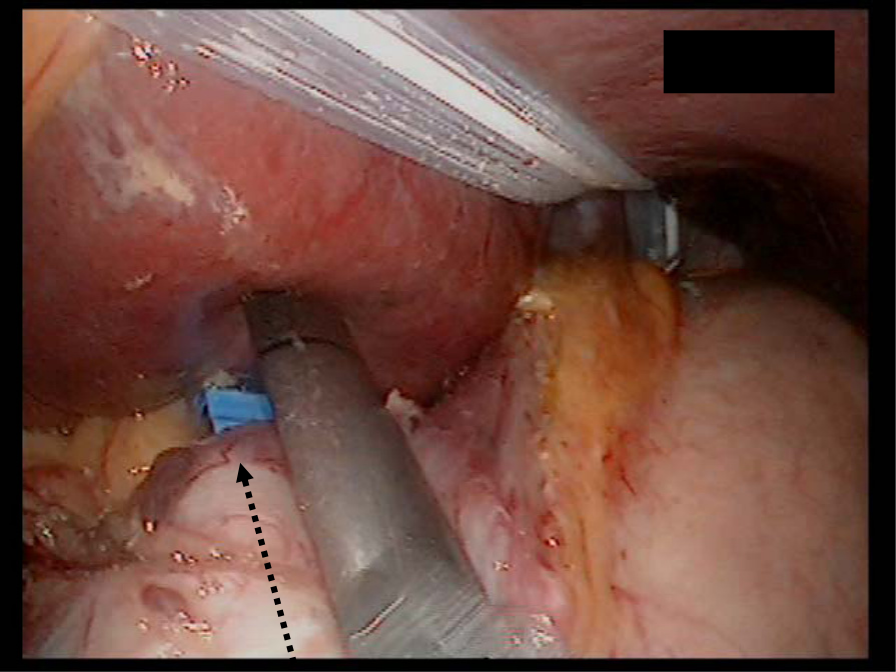
The stomach is separated using two or three linear staples.

Dorsal side lymphadenectomy was performed from the inserted forks to the esophageal side (Figure 7). This method produces a sufficient lymphadenectomy of the ventral side and the dorsal side, in accordance with accepted oncological theory.

We are now able to insert the forks of the endoscopic liner stapler into the stomach from the greater curvature to the lesser curvature. We can remove the distal stomach using the camera port at the umbilical position.

The details of reconstruction of the intracorporeal delta-shaped gastroduodenostomy after LDG are as described by Kanaya *et al*. [2]. All data are presented as mean ± SD. The data were evaluated statistically using the Student’s *t* test, Wilcoxon signed-rank test, log-rank test, and Pearson product–moment correlation coefficient to determine statistical significances. A value of *P* < 0.05 was regarded as indicating statistical significance.

## Results

Patient demographics and clinical histories are shown in Table 1.

**Table 1:** Patient demographics and postoperative outcome

**Characteristics**	**Value (*n*= 14)**	**Range**
Age (year)	67.5 ± 9.1	52 to 78
Sex (male: female)	10:4	
Body mass index (kg/m2)	21.5 ± 2.5	18.2 to 27.2
Blood loss (ml)	39.9 ± 47.4	3 to 190
Operation time (min)	240.2 ± 53.5	167 to 367
Hospital stay (day)	13.3 ± 2.3	8 to 17
Complication (%)	0	

## Discussion

Since laparoscopy-assisted distal gastrectomy (LADG) was first reported by Kitano and colleagues in 1994 [3], the use of laparoscopic gastrectomy for early gastric cancer has been increasing rapidly and gaining in popularity worldwide because it is associated with less wound pain, quicker recovery, and a shorter hospital stay [4].

Many studies have compared the surgical features of LADG and CDG. Some reported longer operation times for LADG than for CDG, as well as longer operation times for LDG than for LADG [5-7]. As a result, it has been concluded that LADG and LDG performed by a skilled and experienced surgeon takes no more time than CDG [8,9]. At present, Billroth I reconstruction is commonly selected for laparoscopic operation by Kanaya [10]. However, intra-abdominal anastomosis is technically difficult. Although some skillful surgeons have presented intra-abdominal hand-sewn techniques, the extra-abdominal approach is now popular for laparoscopic Billroth I gastroduodenostomy, while laparoscopic Roux-Y reconstruction using a circular stapler has also been reported [11-19]. However, this technique is complicated, and an extended incision of about 5 cm at the upper median area is required.

Recently, a number of surgical techniques have reported lymph node dissection at stages 8a, 7, 9, 11p (common hepatic artery, left gastric artery, splenic artery) or stages 6 (right gastroepiploic vein and artery) of in surgical endoscopic meetings. In addition, informational movies related to special surgical technique can be shared on DVD, making them more accessible to endoscopic surgeons. We designed a V-shaped lymph node dissection in LDG.

The techniques used in stages 1 to 3 are generally performed as the final parts of LDG. It is possible to perform stage 1 to 3 lymph node dissection using a small incision.

Moreover, this area is abundant in blood vessels that flow from the stomach to the lesser omentum and bleeding from lesser omentum is often greater than expected. This obstructs the view in the intra-abdominal cavity.

**Figure 8:**
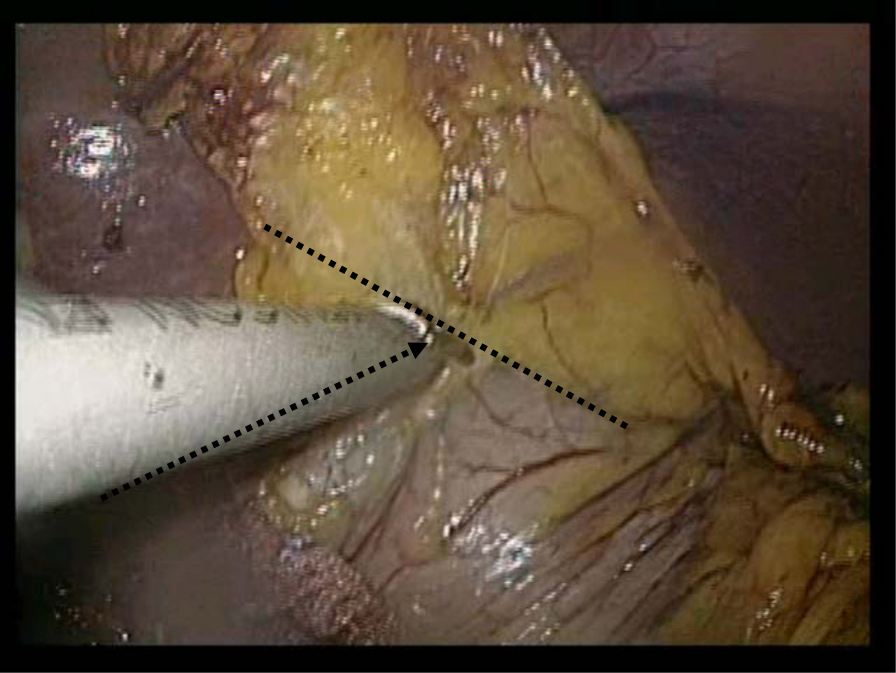
A bad case, demonstrating the need to cross between our forceps and the lesser curvature of stomach wall.

Because we perform the operation from the patient’s right side, our operation intersects squarely between our forceps and lesser curvature of the stomach wall (Figure 8). There is however, a danger of causing injury to the stomach wall and the potential problem of leaving lymphatic tissue in the stomach wall.

Our V-shaped lymph node dissection offers several advantages. Lymph node dissection in the lesser curvature of the stomach is much easier because visibility is improved. When the assistant operator lifts up the V-shaped stomach, the laparoscopic view of ventral and dorsal sides (stages 1 and 3) are also considerably improved. Our method also makes it much easier to cut a straight line along the axis of the dorsal side and along the lesser curvature side against the stomach wall. In addition, forceps placement is easier and more precise. Moreover, the method allows for a smooth, precise operation without the need for additional lymph node dissections.

This method clarifies the separate positions of the stomach using a linear stapler to reduce the number of operations and can therefore be beneficial for cost reduction.

All cases were successfully performed without any intraoperative or postoperative complication. In addition, there was no difference in the pathology lymph node number compared with the advanced gastric cancer when CDG was performed during the same period in our unit (Table 2).

**Table 2:** Characteristics of 45 patients undergoing laparoscopic distal gastrectomy and conventional distal gastrectomy

	**LDG**	**CDG**
Lymph nodes (station 1 to 3)	9.43 ± 3.69	7.65 ± 4.79
Number of cases	14	31
*P* value		0.18

CDG, conventional distal gastrectomy; LDG, laparoscopic distal gastrectomy.

The results suggest this method to be of significant clinical value. Furthermore, we note that LADG is being performed on patients at the early gastric cancer stage in many hospitals in Japan [20].

In this process, further discussion of stages 1 and 3 is required. Currently, it is seen to be a very important area and dissection of stages 1 and 3 need to be classified according to part D1, in accordance with Japanese treatment guidelines.

## Conclusion

We reported a new method of intra-abdominal lymph node dissection for the lesser curvature (stations 1 and 3). In oncological therapy, this technique could be a valuable surgical option for totally laparoscopic surgery.

### Consent

Written informed consent was obtained from the patient for publication of this case report and accompanying images.

## Abbreviations

CDG: conventional distal gastrectomy; LADG: laparoscopic-assisted distal gastrectomy; LDG: laparoscopic distal gastrectomy; TLG: totally laparoscopic gastrectomy.

## Competing interests

The authors declare that they have no competing interests.

## Authors’ contributions

Study conception and design: NM, S-WL. Acquisition of data: MK, NN, CT, YI, S-WL, TT. Analysis and interpretation of data: NM. Drafting of manuscript: NM. Critical revision: NM, S-WL, MK, KK. Supervision: KY. All authors read and approved the final manuscript.
